# A Path Analysis of Relationship between War Trauma, Post - Traumatic Stress and Post Traumatic Growth

**DOI:** 10.12669/pjms.40.10.8897

**Published:** 2024-11

**Authors:** Azra Azeem, Nelofar Kiran

**Affiliations:** 1 Azra Azeem, National Institute of Psychology, Quaid-e-Azam University, Islamabad, Pakistan; 2 Dr. Nelofar Kiran Rauf, National Institute of Psychology, Quaid-e-Azam University, Islamabad, Pakistan

**Keywords:** War Trauma, Post-traumatic Growth, Stress

## Abstract

**Background & Objective:**

Police officials who participated in war on terror were exposed to high frequency of traumatic exposures. Previous research suggests that exposure of war trauma can results in negative changes like stress and positive changes like growth. Our objective was to study the role of post-traumatic stress in relationship between war trauma and post traumatic growth among police officials.

**Methods:**

In this cross-sectional study conducted from January 2019 to December 2022. The sample consisted of 400 police officials (having direct and indirect exposure of war trauma) who participated in war on terror in FATA Pakistan. Non probability purposive sampling technique was used for sample selection. Relationship among trauma, PTS (Post traumatic stress) and PTG (Post traumatic growth) was assessed.

**Results:**

Findings from the present study indicated that stress played vital role in paving pathway from trauma to growth (β =0.08). Study results also confirmed that moderate level of stress is linked with positive changes like posttraumatic growth. R-square change in linear relationship among stress and posttraumatic growth was 0.59 but in curvilinear relationship among stress and posttraumatic growth R-square change was 0.77 which is higher than linear relationship which confirmed the curvilinear relationship between stress and growth and these findings proved that moderate level of stress after war trauma exposure produced higher levels of post traumatic growth. Conditional indirect effect of exposure to trauma on growth is highly significant (***p < .001) so the role of stress as mediator has been confirmed.

**Conclusion:**

The study provided initial evidence that after trauma exposure levels of posttraumatic stress which is experienced play a significant role in the promotion of PTG.

## INTRODUCTION

The effects of traumatic exposure have received much attention in the general population (e.g., survivors of natural disaster, sexual assault) and to a lesser extent in Law enforcement occupations (e.g., police, military and other related departments). Post-traumatic stress and disorder associated with it like posttraumatic stress disorder (PTSD) may be developed after any exposure to tragedies or loss.^1^ DSM 5 has proposed four distinct clusters of post traumatic disorders these clusters are like re-experiencing the tragic event, avoidance, negative cognitions and arousal.^2^ Posttraumatic stress is associated with cardiac abnormality, elevated rates of mortality and suicidal risks. Post-traumatic stress can be result of post reaction to tragic events.^3^ Individuals related to different law enforcement departments like police, military and rescue experience direct and indirect exposure of trauma due to nature of their job. With the goal of better assisting officials who have risks of trauma exposure and posttraumatic stress, enhanced knowledge regarding this psychological response and way out to the development of posttraumatic growth (PTG) is essential.^4^ Studies have confirmed that unexpected, sudden and extreme tragedies may cause post-traumatic stress disorder and anxiety in the exposed population.^5^ Severity of the distress can be measured by the severity of the symptoms like feeling of chronic sadness, worthlessness feelings and loss of interest in interaction with other people.^6^ But all tragedies do not end in negative results some time positive change is experienced after facing traumatic events. However, the factors that may mitigate or promote post-traumatic stress and post-traumatic growth (PTG) after trauma have not yet been authoritatively established.^7^

The most recent and comprehensive work on PTG and PTS by Calhoun & Tedeschi, reiterates that positive changes after trauma do not deny the ongoing distress that may be experienced by some people and therefore, presence of PTG and PTS symptoms together in the after math of trauma are possible.^8^

After experiencing adverse event positive personal changes and bounce back experiences among traumatized population are the core topics of modern research.^9^,^10^ We proposed a framework that describes such interactions based on the PTG revised model presented by Tedeschi. Based on a sample of 400 police officials while performing their duties during war on terror experienced direct and indirect trauma exposure due to terrorist attacks and launching operation against terrorists. We examined the impact of direct and indirect trauma exposure during war on terror on follower police officials, including the mediating effect of posttraumatic stress between trauma and posttraumatic growth in a pathway analysis and have conducted analysis of variance among officials on study variables with different frequencies of trauma exposure. We used the Almost Perfect Measure (i.e., PDS-5 (Posttraumatic stress diagnostic scale) Comprehensive trauma inventory and PTG inventory) along with detailed demographic sheet. Path analysis was used to test the proposed framework. Results confirmed the basic premises of the framework and replicated previous findings of the differential contribution of trauma frequency (based on their severity) to PTG. Study have provided us the evidence that in the process of growth both positive (Growth) and negative effects (PTS) can be experienced together. Inverse U relationship was confirmed between stress and growth which have confirmed that moderate level of stress experienced by police officials contributed significantly to higher levels of PTG.

## METHODS

This cross-sectional study was conducted from January 2019 to December 2022. Sample consisted of 400 police officials with age range 21 years to 58 years. The police officials included in sample were performing their duties in FATA (Federally administered areas of Pakistan) during war on terror. The inclusion criteria of the sample were police officials, exposed to direct and indirect war related traumatic events (like facing life threatening events, witnessing and hearing about injuries and dead bodies, deaths of friends and relatives, witnessing mass graves) during war on terror, and were on post deployment from one year in peaceful areas. Due to exposure of traumatic events during war posttraumatic stress was common in sample which was selected by using non probability purposive sampling technique. Sample size was calculated by open epi calculator and was estimated to be 390 with anticipate frequency of 50%, confidence limit 0.5.

### Ethical approval:

Sample Data were collected after approval of ethical committee of NIP (National Institute of Psychology) (Ref No.: F.No. D-107-3(03), PhD-2019-Admin; dated: January 3, 2019), and permission from the concerned authorities and after taking informed consent from the participants.

Posttraumatic stress was measured by using post-traumatic stress diagnostic scale (PDS-5).^11^ It was translated in Urdu (*α*=0.93). Participants rated their responses on 5-point likret scale. Post traumatic growth inventory^12^ was used as a measure of post traumatic growth. Urdu version was already available. Score of the alpha reliability of the current scale for the sample was 0.91.

Comprehensive trauma inventory^13^ was used for measuring war related trauma exposure severity. Inventory was translated in Urdu language and was culturally adapted. Participants rated their responses on 5-point Likret Scale. The alpha reliability of the scale was .91 for the current research sample.

**Fig.1:**
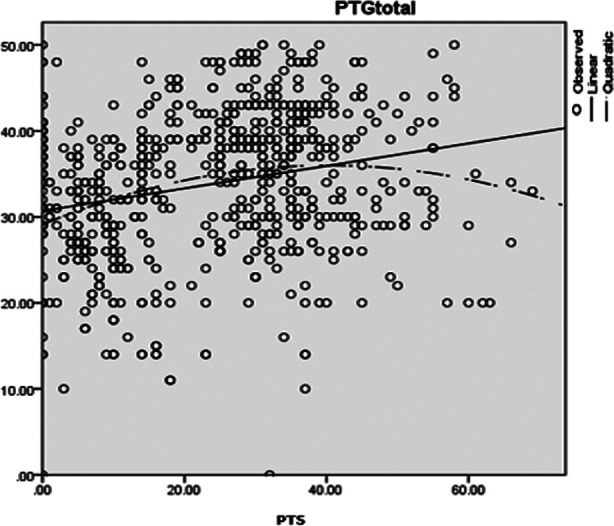
Fig.1: Comparison between Linear and Curvilinear Relationship. Note: PTS = Post traumatic stress, PTG = Posttraumatic growth.

Analysis was done by using AMOS and SPSS version 21. After descriptive analysis of socio demographic variables such as education, age, marital status, duration spent in combat area, frequency of trauma exposure etc. Linear and curvilinear relationship was computed by regression. Mediation was computed to analyze indirect path. ANOVA was computed to see group differences.

## RESULTS

Demographic information of the sample (N=400) was i.e., 200 individuals had experienced direct trauma exposure and 200 had indirect trauma exposure. Sample Mean age was 33.6; SD = 6.3. Married 264 (66%) unmarried individuals were 136(34%) and respectively in the sample. Descriptive analyses of demographic variable are given in Table-I.

**Table-I:** Demographic variables of sample (N=400).

Demographics	f	%
***Trauma Exposure***		
Indirect	200	50 %
Direct	200	50 %
***Frequency of trauma Exposure***		
One time exposure	133	33.25
Two-time exposure	167	41.75%
Three time or more	100	25 %
***Age***		
21-30	100	25 %
31-40	175	43.75%
41-50	100	25 %
51-60	25	6.25%
***Marital Status***		
Married	264	66 %
Un-married	136	34%

Graph between post-traumatic stress which is measured by PDS-5 and post traumatic growth measured by posttraumatic growth inventory had shown a curvilinear relationship. R-square change in linear relationship was 0.59 but in curvilinear relationship, R- square change was 0.77 which was higher than linear relationship and it confirmed the curvilinear relationship between war trauma and growth. This relationship has been confirmed by graph as well. From graph, it is clearly confirmed that growth scores are higher when posttraumatic stress is at moderate level and growth is low when post-traumatic stress is low or high.

The results (Table-II) showed that relationship of exposure to war trauma and growth is conditional indirect and has confirmed the significant mediated role of posttraumatic stress in this relationship. Results has confirmed that indirect relationship as compare to direct relationship have shown higher significance between war trauma and post traumatic growth.

**Table-II:** Mediation of Posttraumatic Stress (N=400).

Models			95% CI			
	***R^2^***	***F***	*β*	***LL***	***UL***	***p***
Constant			30.53	29.38	31.68	.00
Model without mediator Total Effect (CT - PTG)	0.51	38.27	0.72	0.67	0.77	.02
Model with mediator (CT x PTS = PTG)	0.05	22.15	0.11	0.05	0.16	.00
Direct Effect	-	-	0.02	-0.03	0.08	
Indirect Effect	-	-	0.08	0.03	0.12	

*p <.05, **p <.01, ***p < .001.

Results (Table-III) have reported the mean differences for participants with different frequencies of trauma exposure on all study variables. To compute these mean differences, the frequency of trauma exposure was categorized as one time exposure, two-time exposure, three time or more exposure. One way ANOVA revealed significant differences across police officials having different frequencies of trauma exposure on all study variables.

**Table-III:** Analysis of Variance among Police Officials with different frequencies of Trauma Exposure along Study Variables (N=400).

Variables	One-time exposure		2-time exposure		Thrice or more		f	p	eta sq.

	M	SD	M	SD	M	SD			
W-T	30.95	13.4	27.01	12.6	30.14	14.2	147.33	0.00	.338
PDS	29.67	14.45	28.43	13.3	31.87	15.5	108.47	0.00	.319
PTG	33.84	9.02	37.06	7.04	34.40	8.21	30.85	0.00	.117

WT= trauma exposure, PDS= Posttraumatic stress, PTG= Post traumatic growth.

## DISCUSSION

The current study has sought to supplement the literature by understanding impact of trauma, its outcomes and exploring the mediating role of stress and to confirm the path way to post- traumatic growth.^14^,^15^ In addition, the study has undertaken to comprehend the correlates of post-traumatic stress symptoms as impact of trauma for those who have high risk. Study objective was to confirm that what type of relationship exists between war trauma, stress and post traumatic growth, results are consistent with previous literature.^15^ Present study is unique in Pakistan as it has conducted the pathway analysis from war trauma to posttraumatic growth and has studied impact of direct and indirect exposure of war trauma among police officials who had faced trauma exposure while performing their duties during war on terror. Study has investigated under lying relationship between War trauma and PTG and the role of posttraumatic stress in this pathway. Research revealed a quadric relationship between PTS symptom severity and PTG, with. Further present research has confirmed mediated role of stress in this relationship. Moreover, it was analyzed in that what type of relationship (Linear or curvilinear) exist between posttraumatic stress and posttraumatic growth. Curvilinear or quadric relationship can be explained that when two variables show inverted U type relationship it is called Quadric relationship. Results are consistent with previous studies.^16^ Few studies have confirmed the quadric relationship among stress and growth. This type of relationship explains that positive outcome in face of trauma can be experienced when stress is at moderate level while in response to very less stress or extreme stress growth experience is low.^17^

Present study has confirmed that curvilinear (Quadric relationship) relation between posttraumatic stress and posttraumatic growth among police officials was found higher than linear relationship. The curvilinear relationship between posttraumatic stress and posttraumatic growth explains that relationship is week at the low and high levels but strong at moderate level. The results are consistent with the literature. A study conducted on police officials confirmed that growth is experienced at peak at the moderate levels of stress.^18^ Present study has confirmed that growth and stress can be experienced together. Results are consistent with previous studies. Researchers have also reported that PTS positively predicts PTG.^19^ Another research conducted on relationship between PTSS and PTG, however found small and insignificant positive associations between both variables.^20^ More over in order to analyze the pathway from war trauma to posttraumatic growth mediation analysis was conducted. The analysis has confirmed that indirect relationship is more significant which means that posttraumatic stress play mediating role in this relationship. in experiencing positive outcomes after exposure of trauma. Results are consistent with previous research.^21^ For present study, frequency of war trauma exposure was divided into three categories. Like one time exposure, two time trauma exposure and three time exposure. One way ANOVA revealed significant group differences on all study variables among police officials with different frequencies of trauma exposure results are consistent with previous research.^22^

The findings of present study are consistent with the previous research, confirming the function of stress as mediator between posttraumatic growth and war trauma. Research have also proved that moderate level of stress produces higher levels of growth.^23^ Previous research findings have also confirmed the tendency of exposure of traumatic events in law enforcement officials and chances of positive changes after tragidies.^24^

### Limitations & Implications:

Utilization of the self-report measures and use of purposive technique for sample selection are the limitations of the study. With use of random sampling and longitudinal study design provides better qualification to generalization of the findings but due to time limitations and complex sample it was not possible to conduct longitudinal study. Findings have implications for police officials and other law enforcement agencies officials’ fitness programs, psychological intervention planning and health care programs for officials of police and other law enforcement agencies who had faced tragedies while performing their duties during war on terror.

## CONCLUSION

Confirmation of mediating role of stress between trauma and growth through path analysis may help to foster post traumatic growth in individuals by targeting stress and reducing the chances of PTSD. The study high lights the implication of increased clinical efforts to focus on improving the mental health condition of police and other law enforcement agencies officials.
